# Posterior Surgery in the Treatment of Craniovertebral Junction Deformity with Torticollis

**DOI:** 10.1111/os.13324

**Published:** 2022-08-01

**Authors:** Jinpeng Du, Xiangcheng Gao, Yunfei Huang, Xiaobin Yang, Bolong Zheng, Zhongkai Liu, Hua Hui, Lin Gao, Jiayuan Wu, Zhigang Zhao, Baorong He, Liang Yan, Dingjun Hao

**Affiliations:** ^1^ Department of Spine Surgery, Honghui Hospital Xi'an Jiaotong University Xi'an City China; ^2^ Yan'an University Yan'an City China

**Keywords:** Atlas‐dens interval, complications, craniovertebral junction deformity, torticollis, torticollis angle

## Abstract

**Objective:**

To investigate the clinical effect of posterior surgery in the treatment of craniovertebral junction (CVJ) deformities with torticollis and methods for preventing and treating complications in order to obtain a reasonable treatment strategy.

**Methods:**

From January 2007 to December 2017, 78 patients who suffered from CVJ deformities with torticollis treated by posterior surgery were analyzed. The surgical techniques were all posterior correction and fusion to restore the anatomical alignment of the craniovertebral junction. The visual analog score (VAS) and Short Form‐36 (SF‐36) health survey questionnaire were utilized to evaluate preoperative and postoperative neck pain, and changes in the torticollis angle and atlas‐dens interval (ADI) were evaluated through anteroposterior X‐ray and computed tomography. Intra‐ and postoperative complications were all recorded. One‐way ANOVA, LSD‐t test, and χ^2^ test were performed to evaluate the difference between the preoperative and postoperative data.

**Results:**

The mean follow‐up time was 37.4 ± 15.7 months, the average operation time was 115.6 ± 12.8 min, and the average blood loss was 170.8 ± 26.3 mL. According to the deformity site, the range of posterior correction and fusion was as follows: 38 cases of C_1_–C_2_, 33 cases of C_0_–C_2_, and seven cases of C_0_–C_3_. The preoperative SF‐36, VAS, torticollis angle, and ADI were 42.6 ± 8.8, 4.8 ± 1.1, 37.2 ± 11.2°, and 4.9 ± 2.3 mm, respectively. The difference was significant at 3 months post operation (*p* < 0.05), and there was no significant difference at the final follow‐up compared with 3 months post operation (*p* > 0.05).

**Conclusion:**

It can objectively achieve favorable correction and satisfactory clinical effects under posterior correction and fixation for CVJ deformities with torticollis. Intra‐ and postoperative complications can be settled by proper management.

## Graphical abstract

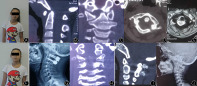

## Introduction

The craniovertebral junction (CVJ) refers to the anatomical functional complex comprising the occipital bone, atlas, and axis and their surrounding ligaments, blood vessels, nerves, and other tissues around the foramen magnum^1^. The osseous dysplasia or structural abnormalities that constitute this complex will cause malformations in the appearance of the patients. In more serious cases, it may cause nerve and vascular damage, resulting in complex clinical manifestations^2^. The many causes of CVJ deformities can be divided into congenital and acquired and can be seen alone or in combination. Its manifestations include flat skull base, skull base depression, atlantooccipital fusion, free odontoid process, atlantoaxial dislocation, and C_2_‐_3_ vertebral body fusion as well as nervous system malformations such as cerebellar tonsillar hernia and syringomyelia^3^. CVJ deformities are often not a single form of the disease and are often accompanied by multiple forms of malformation. However, a class of patients only show appearance deformities but have no nervous system symptoms in the clinic. The reason for this kind of patient is torticollis. Torticollis often presents as the head or neck in a tilted and rotated position, which is a clinical manifestation, not a specific diagnosis. At present, the surgical approaches for CVJ deformities with torticollis include the transoral combined anterior and posterior approach, simple anterior approach, and simple posterior approach. Posterior surgery is the main treatment because of its simple operation and high rate of bone grafting and fusion, which can achieve good clinical results^1^. Torticollis is most common in children and has an estimated incidence of 0.3% to 1.9%^4^. To date, there are more than 80 causes of torticollis, including a variety of congenital and acquired diseases^5^. The most common cause is muscular torticollis. As long as it is found in time, the prognosis is good^6^. However, bony torticollis is difficult to find from the appearance at birth or before 5 years old, especially in patients without neurological symptoms, but torticollis gradually manifests at approximately 8 to 10 years of age with growth and development, and the face already shows asymmetry at this time. Some of these patients have lower cervical vertebral deformities, and the difficulty of operation is relatively low, while the incidence of other patients with craniocervical junction deformities is very low, easily causing misdiagnosis or missed diagnosis. However, even if the diagnosis is clear, it is difficult to unify the treatment. At present, there are few studies about CVJ deformities with simple torticollis, and the incidence is low; thus, clinicians have limited understanding of the disease and a lack of experience in diagnosis and treatment. Therefore, we reviewed and analyzed patients with CVJ deformities complicated with bony torticollis who have been treated in recent years. The purpose of this study is as follows: (i) to summarize the clinical characteristics and treatment of CVJ deformities complicated with bony torticollis and (ii) to analyze the postoperative complications and their causes.

## Patients and Methods

### Patient Demographics

The study was conducted in agreement with the Declaration of Helsinki and was approved by the Ethics Committee of the Honghui Hospital in China (No. 2011086). All patients provided written informed consent. From January 2007 to December 2017, 78 patients with CVJ deformities with bony torticollis were treated. The clinical manifestations of all patients were torticollis, where the head was tilted to one side, the neck was stiff, and the bilateral facial development of some patients was asymmetrical.

### Inclusion and Exclusion Criteria

Inclusion criteria: (i) torticollis patients with CVJ deformities (short neck deformity, skull base depression, hemivertebra deformity, atlanto‐occipital fusion, and dentate paroxysmal malformation); (ii) neck stiffness or pain with or without bilateral facial asymmetry; (iii) no neurological symptoms; (iv) patients treated with posterior orthopaedic fixation and fusion; and (v) follow‐up time ≥3 years.

Exclusion criteria: (i) cerebellar tonsillar hernia deformity, syringomyelia, medulla oblongata or cervical spinal cord compression with neurological symptoms; (ii) craniocervical junction deformities caused by trauma, infection, tumor, or other osteopathy; and (iii) muscular torticollis.

## Surgical Procedures

### Posture and Anesthesia

All patients used general anesthesia, were placed in a prone position, and maintained axial traction (traction weight is 1/6 of body weight) in the process of turning over to prevent spinal cord injury due to atlantoaxial joint activity. The forehead was placed on the head frame of the operating bed, and the cervical vertebra was kept in a neutral position under the action of skull traction. The deformities of all patients were corrected to varying degrees under traction.

### Approach

The posterior occipitocervical incision from the nuchal line to the C_3_ spinous process was taken to separate the suboccipital minor muscle group from the midline to expose the posterior arch of the atlantoaxial vertebra.

### Surgical Method

Torticollis caused by C_1_–C_2_ malformation: If the deformity was located in the atlantoaxial vertebra, atlantoaxial orthopaedic fixation was chosen. Atlas pedicle screws and axial pedicle screws were the first choices. The specific methods of setting nails were as follows^7^: the entry point of the pedicle of the atlas was chosen at the intersection of 18–20 mm near the midpoint of the posterior tubercle of the atlas and 3 mm above the inferior edge of the posterior arch. The cortical bone of the atlas posterior arch was removed by grinding, and the screw path was prepared along the atlas pedicle with an inner inclination angle of 0°–10°. The upper inclination angle was approximately 5°, and the depth was approximately 30 mm. The screw was tapped and placed, and the same operation was performed on the opposite side. On the axis, the midpoint of the root of the articular process is the entry point, the cortical bone of the entry point was removed by grinding, and the electric drill gradually reached the pedicle along the upper and inner cortex of the isthmus. At this time, the internal inclination angle of the nail path was approximately 15°, and the upper inclination angle was approximately 30°, the screw was tapped and placed, and the same operation was performed on the opposite side. The corresponding length of the titanium rod was selected, the rod was prebent into a certain curvature, the tail end of the atlantoaxial fixation screw was connected, and the rod and nail were locked with the top wire, lifted, stretched, or held according to different surgical requirements to make the atlantoaxial joint as close to the anatomical state as possible. If one side of the atlantoaxial lateral mass was fused, a grinding drill was used to grind open the fused joint surface to facilitate reduction. Bone was taken from the ilium and placed between the posterior arch of the atlantoaxial vertebra, and the cancellous bone was spread around it.

Torticollis caused by C_0_– C_1_ malformation: occipitocervical orthopaedic fixation was selected for these deformities. After exposing the occipital scale, the axial vertebral arch, and the posterior edge of the foramen magnum, the pedicle screws were placed in the isthmus of the axial arch in the same way as the atlantoaxial orthopaedic fixation. If the C_2_–C_3_ vertebral body was not segmented, lateral mass screws or pedicle screws were placed on both sides of C_3_. The occipital screw was placed on both sides of the external occipital eminence as close to the midline as possible. The titanium rod of suitable length was prebent into a certain curvature, and the top wire was rotated into the C_2_ or C_3_ pedicle screw tail. In the process of tightening the top wire, the degree of prebending of the titanium rod was used to fully restore the atlantooccipital or atlantoaxial joint.

Torticollis caused by C_0_–C_2_ malformation: the operation is basically similar to torticollis caused by C_0_–C_1_ deformity, and occipitocervical orthopaedic fixation was also selected. The difference is that in this kind of malformation the anatomical relationship of the atlantoaxial vertebra should first be restored, and then the anatomical relationship of the atlantooccipital joint should be restored; this is suitable for patients with occipital atlantoaxial rotational fixation and atlantoaxial malformation with atlantooccipital fusion.

## Postoperative Treatment

Skull traction was removed immediately postoperatively, the incision drainage tube was removed 24 h later, and antibiotics were used no more than 24 h postoperatively. After removing the drainage tube, the patient could sit up or get out of bed under the protection of a neck brace. According to the bone fusion, the cervical brace was removed for rehabilitation training after 3 months, and physical activities were avoided within 3 months. The postoperative reexamination was specifically supervised by the research assistant. The patients were reexamined every 3 months within 1 year and every 6 months after 1 year. The patients were followed up for at least 3 years.

## Evaluation

### Radiographic Measurement

X‐ray, CT, and MRI examinations were performed routinely preoperatively. After the operation, the patients were examined by anterior and lateral cervical X‐ray and CT, the orthopaedic and fusion were evaluated, and the position of the screw was observed.

#### Torticollis Angle

The torticollis angle was used to evaluate the degree of torticollis, and it was defined as the angle between the bilateral mastoid tip line on the neutral positive X‐ray and the extension line of the C2 inferior endplate.

#### Atlas‐Dens Interval

**Fig. 1:**
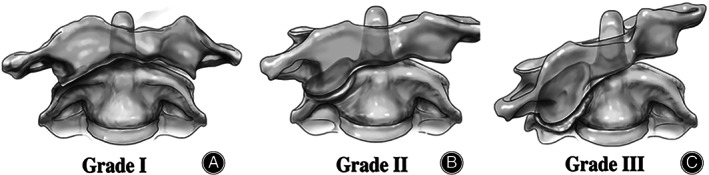
Ishii grading system for atlantoaxial rotation and fixation. (A) Grade I, no articular surface deformity, no lateral angulation; (B) Grade II, articular surface deformity, lateral angle <20°; (C) Grade III, articular surface deformity, lateral angle >20°.

The atlas‐dens interval (ADI) was used to evaluate the degree of atlantoaxial joint dislocation, and it was defined as the distance between the posterior cortex of the anterior arch of the atlas and the anterior cortex of the kyphosis^8^. The characteristics of the articular surface of the bilateral mass fixed with atlantoaxial rotation were divided into three grades according to the Ishii grading system^9^ by CT three‐dimensional imaging (Figure 1).

### Assessment of Clinical Efficacy

The cervical visual analog scale (VAS) and Short Form‐36 (SF‐36) were used to evaluate the changes and clinical effects of neck pain pre operation and post operation.

#### Cervical Visual Analog Scale

The VAS score system is used in the social and behavioral sciences to measure a patient's degree of pain. Patients were asked to rate their pain on a scale of 0 to 10. The VAS pain scoring standard was as follows: 0 means painless; 1–3 means mild pain that the patient could endure; 4–6 means the patient was in pain that could be endured and was able to sleep; and 7–10 means the patient had intense pain and was unable to tolerate the pain. A higher score means greater pain intensity.

#### Short Form‐36

SF‐36 is the most widely used health‐related quality of life (QOL) instrument in the world and has been subjected to extensive testing for reliability and validity. It assesses a broader range of health concerns than skin‐specific measures by evaluating the following eight domains: physical function (PF), role limits owing to physical problems (RP), bodily pain (BP), general health (GH), social functioning, vitality (VT), role limits owing to emotional problems (RE), and mental health (MH). These eight domains are further aggregated into a Physical Component Summary score (PF, RP, BP, and GH) and a Mental Component Summary (MCS) score (social functioning, VT, RE, and MH). A higher score indicates better QOL.

### Statistical Methods

SPSS 19.0 software (IBM, Armonk, NY) was used for statistical analysis, and the measurement data were expressed as the mean ± standard deviation. One‐way ANOVA was used to compare the preoperative and postoperative data, and then the LSD‐t test was performed. The counting data were tested by a χ^2^ test. The difference was statistically significant at *p* < 0.05.

## Results

### Follow‐up Results

All patients were followed up for more than 3 years, with an average of 37.4 ± 15.7 months. X‐ray, CT, and clinical examinations were performed in all patients at 3 months after surgery and at the last follow‐up. The torticollis angle and ADI 3 months after surgery and at the last follow‐up were measured based on radiation imaging. The SF‐36 scale and neck VAS at 3 months after surgery and the last follow‐up were evaluated.

### Radiographic Improvement

The self‐reported facial asymmetry of 18 patients (23.1%) was improved compared with that preoperatively. Except for four patients who showed bony fusion on CT 9 months postoperatively, all other patients achieved bony fusion 3–6 months postoperatively.

### Clinical Improvement

The preoperative SF‐36 score, VAS score, torticollis angle, and ADI were 42.6 ± 8.8, 4.8 ± 1.1, 37.2° ± 11.2°, and 4.9 ± 2.3 mm, respectively. The difference was significant at 3 months after the operation (*p* < 0.05), and there was no significant difference between the last follow‐up and 3 months postoperatively (*p* > 0.05) (Table 1). The results showed significant differences in the SF‐36 score, VAS score, torticollis angle, and ADI among the different Ishii grades before the operation (*p* < 0.05), but there was no significant difference in the SF‐36 score, VAS score, torticollis angle, and ADI at 3 months post operation and the last follow‐up (*p* > 0.05) (Table 2).

**Table 1:** General information of the patients

Parameters	Value
Age (years)	11.5 ± 2.6
Gender	
Male	24
Female	54
Clinical presentation	
Neck pain	64
Torticollis (left/right)	36/42
Limited rotation of head and neck	56
Symptoms of basilar artery ischemia	32
Atlas‐dens interval (mm)	4.9 ± 2.3
Major deformities	
Atlantoaxial rotational fixation	31
Atlantoaxial rotational fixation with right atlantoaxial joint fusion	5
Atlantooccipital joint developmental deformities	4
Asymmetrical lateral mass development	7
Atlantoaxial arch right segmental insufficiency	18
Occipital atlantoaxial rotation and fixation	13
Combined deformity	
C_2_–C_3_ fusion	4
Atlantooccipital fusion	17
Odontoid dysplasia	12
Double rib	5
Cervical rib	6
High scapula	9
None	25
Other deformities	
Syringomyelia	10
Diastematomyelia	9
Tethered cord	7
Congenital absence of kidney	8
Polycystic kidney	11
Hypertrophic cardiomyopathy	4
Mitral valve prolapse	3
Ear malformations	6
Gastrointestinal malformations	4
Eye malformations	4
Ishii classification	
Grade I	5
Grade II	42
Grade III	31
Course of disease (months)	9.2 ± 2.3
Pre‐admission treatment	
With braces	57
Massage and manual reduction	10
Skull traction	11
Mode of operation	
C_1_–C_2_ fixation and fusion	38
C_0_–C_2_ fixation and fusion	33
C_0_–C_3_ fixation and fusion	7
Operation time (min)	115.6 ± 12.8
Intraoperative blood loss (mL)	170.8 ± 26.3
Length of stay (days)	12.7 ± 3.9

**Table 2:** Results of preoperative, postoperative 3 months and final follow‐up

Index	Pre operation	Post operation 3 months	Last follow‐up
SF‐36 (Points,^−^x ± s)	42.6 ± 8.8	–	51.8 ± 9.7^**b**^
VAS (Points,^−^x ± s)	4.8 ± 1.1	1.1 ± 0.2^**a**^	0.9 ± 0.3^**b**^
Torticollis angle (°,^−^x ± s)	37.2 ± 11.2	10.3 ± 3.7^**a**^	12.1 ± 2.9^**b**^
ADI (mm,^−^x ± s)	4.9 ± 2.3	2.3 ± 0.6^**a**^	2.1 ± 0.5^**b**^

There was significant difference between 3 months after operation and before operation (^a^ *p* <0.05), and between the last follow‐up and 3 months after operation (^b^ *p* >0.05).

### General Results

**Fig. 2:**
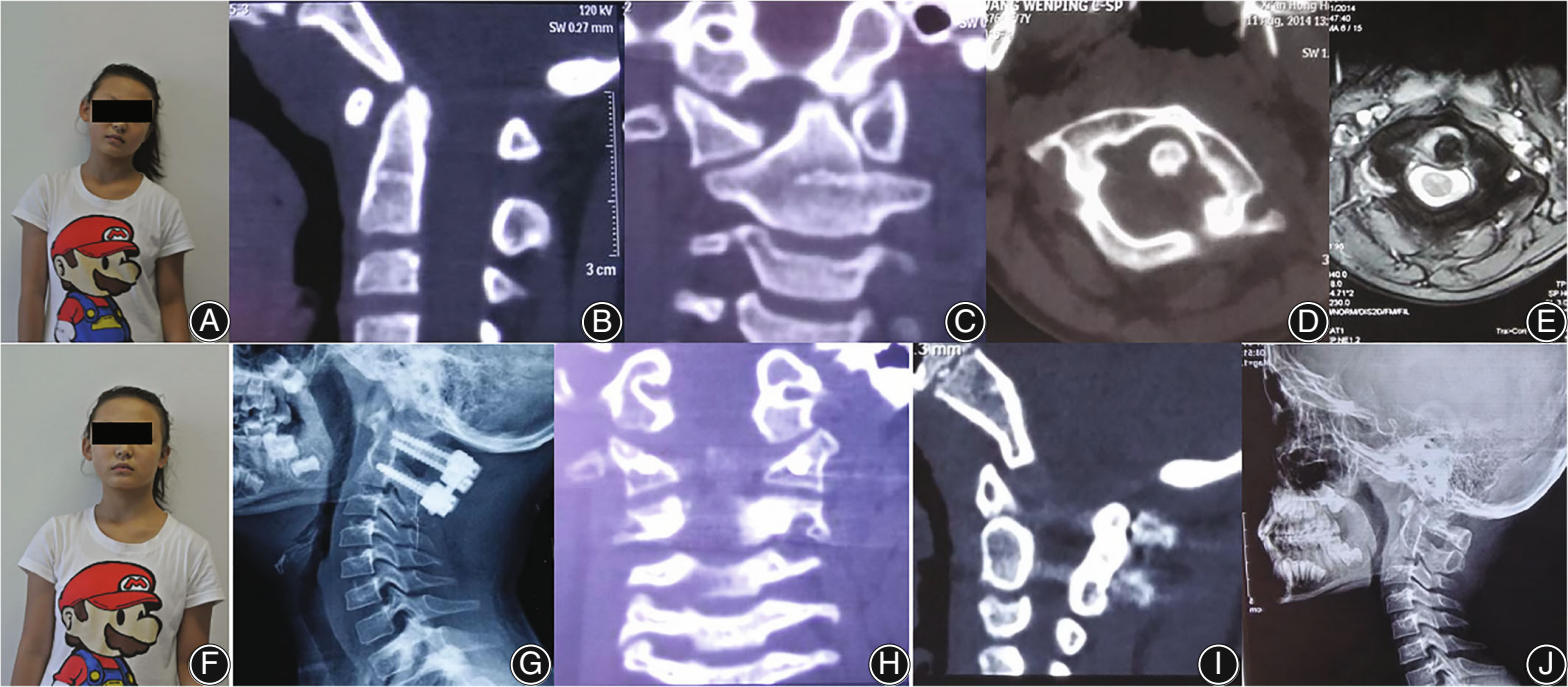
The female, 11 years old, was found to be torticollis for 5 months. (A) The patient showed left torticollis. (B–D) Preoperative CT suggested rotational fixation of atlantoaxial vertebrae, and ADI was 4.3 mm. (E) Axial MRI indicates relaxation of transverse ligament. (F) Postoperative torticollis correction. (G) Post operation 3 days, lateral X‐ray showed that ADI decreased to 2.3mm. (H) Coronal CT post operation 3 months showed that the orthopaedics were maintained well. (I) Post operation 1 year, CT showed bone graft fusion. (J) Post operation one and a half years, there was no loss after internal fixation.

**Fig. 3:**
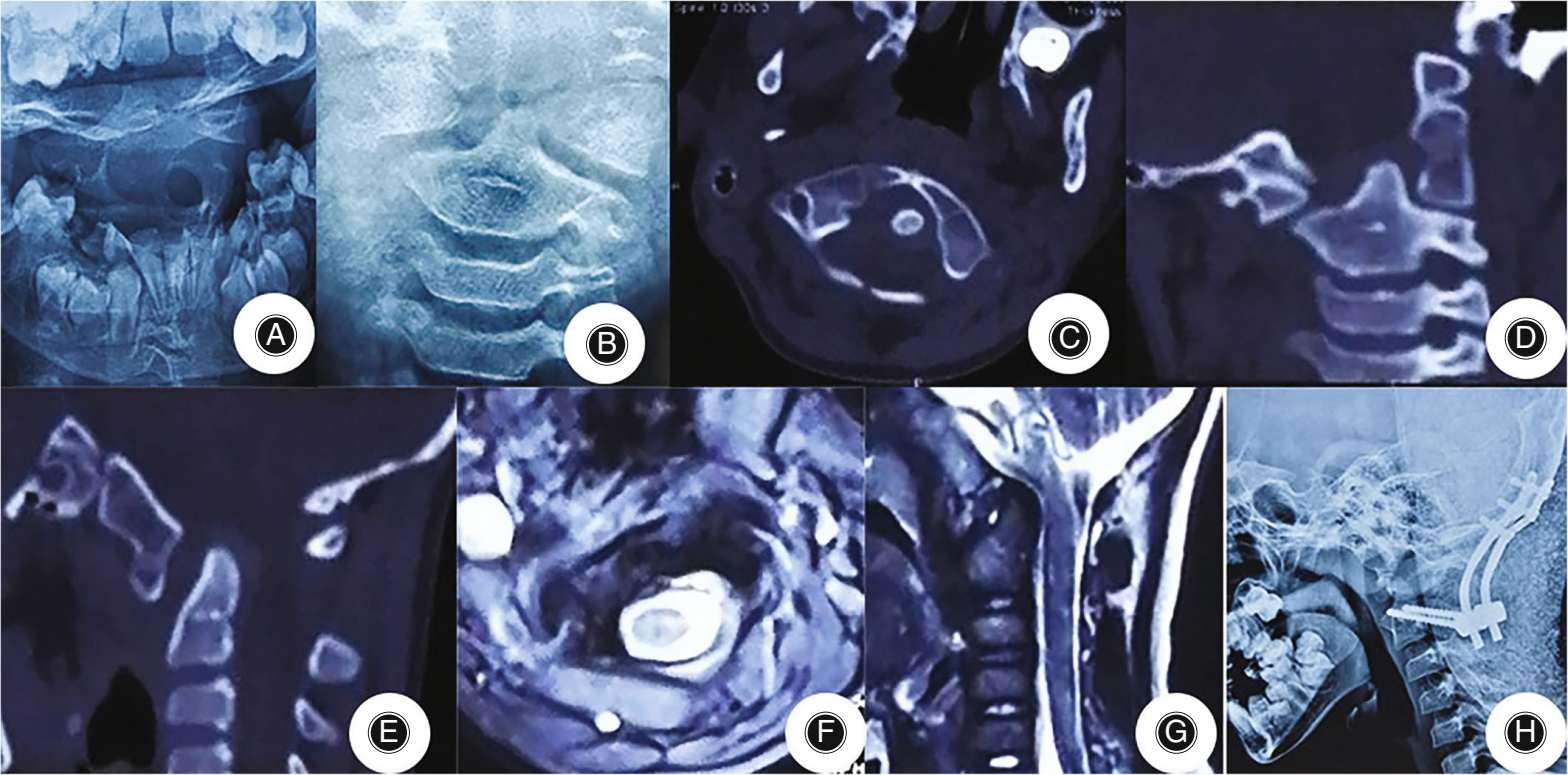
The patient, male, 8 years old. (A) Mouth‐opening position. (B) Anterior position X‐ray films of cervical vertebrae pre operation. (C–E) Preoperative CT images showed rotational fixation of occipital atlantoaxial, and ADI was 4.6mm. (F) Axial MRI indicated relaxation of transverse ligament. (G) Sagittal MRI shows kyphosis of C2 vertebrae and corresponding horizontal spinal canal stenosis. (H) Post operation 7 days, lateral image of the cervical spine showed good reduction and internal fixation and ADI decreased to 2.4 mm.

A total of 78 patients who met the criteria were included. There were 24 males and 54 females with an average age of 11.5 ± 2.6 years. The average course of disease before admission was 9.2 ± 2.3 months, including right torticollis in 42 cases and left torticollis in 36 cases. The average ADI is 4.9 ± 2.3 mm. In terms of major deformities, 31 cases were atlantoaxial rotational fixation, five cases were atlantoaxial rotational fixation with right atlantoaxial joint fusion, four cases were atlantooccipital joint developmental deformities, seven cases were asymmetrical lateral mass development, 18 cases were atlantoaxial arch right segmental insufficiency, and 13 cases were occipital atlantoaxial rotation and fixation. The deformities associated with occipital atlantoaxial rotation and fixation included C_2_–C_3_ fusion (four cases), atlantooccipital fusion (17 cases), odontoid dysplasia (12 cases), double‐rib (five cases), cervical rib (six cases), and high scapula (nine cases). In terms of Ishii grade^9^, five cases were grade I, 42 cases were grade II, and 31 cases were grade III. Among them, 26 cases were complicated with intraspinal malformations, including 10 cases of syringomyelia, nine cases of diastematomyelia, and seven cases of tethered cord; 19 cases were complicated with urinary system malformations (eight cases of congenital renal agenesis and 11 cases of polycystic kidney); seven cases were complicated with heart malformations (four cases of hypertrophic cardiomyopathy and three cases of mitral valve prolapse); and six cases were complicated with ear malformations. Four cases were complicated with gastrointestinal deformities, and four cases had ocular malformations. A summary of the cases is shown in Table 3. All 78 patients were treated with posterior orthopaedic fixation and fusion. There was no spinal cord or vertebral artery injury. The surgical procedures included C_1_–C_2_ fixation fusion (*n* = 38), C_0_–C_2_ fixation fusion (*n* = 33), and C_0_–C_3_ fixation fusion (*n* = 7), all of which were performed through a one‐stage posterior approach without anterior oral release. The operation time was 115.6 ± 12.8 min, and the intraoperative blood loss was 170.8 ± 26.3 mL. The hospital stay was 12.7 ± 3.9 days. Typical cases are shown in Figures 2 and 3.

**Table 3:** Results of pre‐operation, 3 months after operation, and final follow‐up under different Ishii grades

	Pre operation				Post operation 3 months				Last follow‐up			
Follow‐up	SF‐36 (score)	VAS (score)	Torticollis angle (°)	ADI (mm)	SF‐36 (score)	VAS (score)	Torticollis angle (°)	ADI (mm)	SF‐36 (score)	VAS (score)	Torticollis angle (°)	ADI (mm)
Grade I (5)	40.3 ± 5.5	5.4 ± 2.1	14.3 ± 3.2	3.2 ± 2.3	‐‐	1.2 ± 0.4	10.5 ± 3.6	2.1 ± 0.6	50.2 ± 2.7	0.9 ± 0.3	10.6 ± 2.4	1.9 ± 0.6
Grade II (42)	39.6 ± 7.8	5.3 ± 1.7	16.4 ± 3.2	4.5 ± 2.6	‐‐	1.3 ± 0.1	9.3 ± 4.7	2.0 ± 0.2	47.9 ± 8.8	0.9 ± 0.1	11.5 ± 2.5	1.8 ± 0.9
Grade III (31)	38.6 ± 8.9	5.9 ± 1.9	30.2 ± 10.2	5.1 ± 2.2	‐‐	1.3 ± 0.5	11.3 ± 2.7	2.0 ± 0.6	51.8 ± 6.7	0.8 ± 0.2	12.1 ± 2.7	1.9 ± 0.7
*p* value	<0.0001	<0.0001	<0.0001	<0.0001	‐‐	0.121	0.067	0.098	0.230	0.059	0.702	0.081

### Complications

In terms of complications, dural rupture occurred in three cases during lysis, and it was blocked with biological protein gel. A small amount of cerebrospinal fluid leakage occurred after the operation, and the drainage tube was routinely removed 48 h post operation. Bone cleavage occurred in two cases when the nail was placed in the axial isthmus and was fixed with a halo vest post operation. Two months post operation, one occipitocervical fixator loosened on the occipital side in four cases and was fixed with a headband vest. Nine months post operation, CT suggested bone healing.

## Discussion

Based on our results, we can conclude that for patients with CVJ deformity associated with torticollis, posterior correction and fixation can achieve favorable correction and satisfactory clinical effects. In patients with torticollis complicated with CVJ deformities, the common diseases were atlantoaxial rotation fixation, occipital atlantoaxial rotation fixation, atlantoaxial lateral mass development asymmetry, atlantooccipital joint deformity, and atlantoaxial arch unilateral segmentation. Among them, atlantoaxial rotation fixation was the most common, and this has also been reported in previous literature^10^, ^11^. Most of the causes were congenital atlantoaxial lateral mass dysplasia, which leads to atlantoaxial rotational subluxation; it is reversible at first, but the atlantoaxial joint in the state of atlantoaxial subluxation for a long time will lead to further deformation of the atlantoaxial joint. Due to the strong shaping ability of bone joints in children, grade II and III states in the Ishii grading system^9^ manifest in a short time due to the hypertrophy of the articular capsule and the formation of fibrous tissue in and around the joint. The atlas is in a fixed subluxation, which even leads to the fusion of the lateral mass on one side of the atlantoaxial side; thus, the torticollis is gradually obvious, accompanied by the stiffness of the neck. The neck muscles are tense for a long time, and the symptoms of neck pain easily occur. The VAS score of neck pain in this group decreased from 4.8 ± 1.1 preoperatively to 1.1 ± 0.2 3 months postoperatively. The preoperative torticollis angle was 37.2° ± 11.2°, the ADI was 4.9 ± 2.3 mm, and they decreased to 10.3° ± 3.7° and 2.3 ± 0.6 mm, respectively, 3 months postoperatively. The difference was significant, indicating that posterior orthopaedic fixation and fusion can effectively relieve neck pain and achieve a good clinical effect. The results show significant differences in the SF‐36 score, VAS score, torticollis angle, and ADI among the different Ishii grades before the operation, but there was no significant difference in the SF‐36 score, VAS score, torticollis angle, and ADI at 3 months and the last follow‐up post operation. Therefore, the selection of appropriate treatment for CVJ malformation with bony torticollis with different Ishii grades can achieve satisfactory results.

### Analysis of Clinical Characteristics

Atlantoaxial rotatory fixation is relatively familiar to spinal surgeons, but occipital atlantoaxial rotation fixation is relatively rare. Previous literature has mostly reported individual cases^12^, ^13^, ^14^, ^15^, ^16^, ^17^. The first case was reported by Washington *et al*. in 1959^17^, and the authors believed that in patients with partially uncorrectable atlantoaxial rotation and fixation, compensatory rotation of the occipital bone in the opposite direction results in occipital atlantoaxial rotation and fixation. Pang *et al*. found that the rotation of the occipital relative to the atlas increased significantly if the atlantoaxial rotation was fixed for more than 3 months^18^. In the above study, in patients with atlantoaxial rotation fixation within 3 months, the rotation of occipital bone relative to the atlas was 9.1° ± 2.1°; meanwhile, in patients with atlantoaxial rotation fixation for more than 3 months, the rotation of occipital bone relative to the atlas was 31.2° ± 6.9°, and the difference was statistically significant. Pang *et al*.^18^ found that 64.5% of the compensation came from the atlantooccipital joint, and only 35.5% came from the lower cervical spine in patients with atlantoaxial rotational fixation for more than 3 months. The risk factors for chronic atlantoaxial rotational fixation include ligament and joint capsule contracture, synovial articular fibrous tissue formation, atlantoaxial articular surface bony fusion, and atlantoaxial articular surface deformity^9^.

This group of patients was also complicated with other types of CVJ deformities, such as asymmetry of the lateral mass of the axis, developmental deformity of the atlantooccipital joint, and unilateral segmental insufficiency of the atlantoaxial arch. There are many kinds of malformations in the CVJ, including bony malformations such as a flat skull base and depressed skull base as well as nervous system malformations such as cerebellar tonsillar hernia and syringomyelia^19^, but patients with these malformations often show neurological symptoms. However, none of the patients included in this group had neurological symptoms, only simple bony malformation. During intraoperative exposure, the high‐speed drill may damage the venous plexus or vertebral artery. In addition, screw implantation in the upper cervical region may damage the spinal cord and vertebral artery. For asymmetrical development of the axial lateral mass, the atlas and axial pedicle screws should be placed during the operation, and the screws on the less‐developed side of the axial lateral mass should be properly loosened and then fixed and fused. For atlantooccipital joint deformities, most cases were reducible atlantooccipital malformations; occipitocervical fixation and fusion were performed after traction reduction under general anesthesia. For right segmental incompleteness of the atlantoaxial arch, a grinding drill was used to open the posterior arch during the operation. The screw‐rod system was used to open the fusion side and fix the fusion. Therefore, in the following treatment section, we focus on the first two more complex deformities.

### Treatment Protocol Analysis

No preoperative skull traction was performed in all cases, and only skull traction with six body weights was performed after general anesthesia. Reducible atlantoaxial rotation fixation only requires atlantoaxial fusion under traction reduction. For irreducible atlantoaxial rotation fixation, reduction was performed through a screw system. Oda^20^ used posterior reduction, fixation, and fusion for the first time in 1999; and it then gradually developed into a standard method once the curative effect was clear. Some scholars use the anterior transoral approach to release atlantoaxial vertebrae when necessary. Wang Chao and others^21^ believe that a better reduction effect can be obtained by anterior release combined with posterior fixation for irreducible atlantoaxial rotational fixation. However, in this study, four patients showed atlantoaxial rotational fixation combined with right atlantoaxial joint fusion. After grinding the fused joint surface with a grinding drill, the fused joint surface was reduced by a nail‐rod system, and good correction was obtained. The reasons for not choosing the anterior approach for oral release are as follows: (i) to avoid the risk of infection during oral surgery; and (ii) atlantoaxial rotation fixation is generally characterized by the fusion of unilateral lateral mass joints, whereas there is no bony fusion between the anterior arch of the atlas and the odontoid process; thus, the fused lateral mass joints can be ground through the posterior approach.

For occipital atlantoaxial rotatory fixation, cervical occipital orthopaedic fixation was performed in this study, but there were two cases of occipitocervical fixation^13^, ^14^ and four cases of atlantoaxial fixation reported in previous literature^12^, ^15^, ^16^, ^17^. This suggests that with the correction of atlantoaxial deformities, the secondary rotational displacement of occipital bone relative to the atlas can also be corrected by itself. If this theory is correct, then one fusion segment can be reduced, and the limitation of the range of motion of the cervical spine can be reduced, but the previous literature reports do not provide long‐term follow‐up data for more than 1 year; thus, the curative effect cannot be compared. Before valid data were available to support the above conclusion, we still chose occipitocervical fixation to ensure the stability of the atlanto‐occipital joint and avoid the risk of potential rheumatoid arthritis and instability of the atlanto‐occipital joint in some patients. In this study, except for four patients with loss of correction due to occipital screw loosening, all patients achieved neutral correction of the head, and CT showed bony fusion at the follow‐up 3 to 6 months post operation.

The cable binding technique was not used in this group of cases. Although these methods are simple and timesaving, their mechanical effects are not as stable as those of rigid nail‐rod systems; however, they are still suitable for patients with complex deformities who are unable to undergo screw fixation. Screw‐rod system fixation can provide immediate stability post operation, patients can perform early rehabilitation exercises, and pedicle screws are recommended as much as possible when the atlas pedicle can be placed with screws. For screw‐rod system fixation of complex deformities, to ensure that the screw implantation position is accurate and the depth is appropriate, especially without causing vertebral artery injury, the application of intraoperative CT navigation or robot‐assisted positioning can effectively increase the accuracy of screw placement^22^, ^23^, ^24^. Intraoperative spinal cord and vertebral artery injury can be seen in the relevant literature. In our study, (i) through sufficient preoperative assessment, spinal cord stimulation was reduced as much as possible in the process of decompression, and the inner wall could be detected by a nerve hook before nail placement; (ii) for protection of the vertebral artery, a 3D model of the atlantoaxial vertebra preoperatively should be printed to fully evaluate the course of the vertebral artery and avoid vertebral artery injury; (iii) for the problem of venous plexus bleeding, the author's experience is to avoid electrocoagulation and use gel sponges and brain cotton tablets to stop bleeding as much as possible.

### Complications Analysis

In terms of intraoperative and postoperative complications, dural rupture occurred in three cases because the dura mater was damaged when the lateral mass joint was released with a grinding drill, but the tube was extubated 24 h after operation with bioprotein gel. Bone splitting occurred in two cases during the placement of the nail in the axial isthmus due to the high span of the vertebral artery on this side, and the open cone of the vertebral artery was too eccentric to avoid injury. The occipital screw was loosened in four cases, which may be due to the thin occipital bone and the limited holding force of the screw, but bone healing was achieved 9 months postoperatively.

## Limitations

This study has the following shortcomings. First, this study is a retrospective study without a control group and statistical comparison; thus, the level of evidence is not sufficiently high. Therefore, a large sample of prospective randomized studies are needed to support our findings. Second, because of the low incidence of this kind of disease, the number of cases is small, the statistical effectiveness is limited, and it is difficult to find some complications with a low incidence. In summary, despite these limitations, for craniocervical junction deformities with bony torticollis, posterior orthopaedic fixation and fusion can obtain good deformity correction and definite clinical effects.

## Conclusion

Our results suggest that posterior surgery in treating CVJ deformities with torticollis can achieve favorable correction and satisfactory clinical effects. If the deformity involves the atlas and axis, atlantoaxial correction and fusion are chosen; if the deformity involves the atlas, axis, and occiput or purely involves the axis and occiput, occipitocervical correction and fusion are chosen. Furthermore, intra‐ and postoperative complications can also be settled by proper management. In summary, posterior surgery is a relatively safe and effective technique, which should be recommended for the use in patients with CVJ deformities with torticollis.

## Conflicts of Interest

There are no conflicts of interest.

## Funding Information

The General Project from the Xi'an Municipal Health Committee (2020yb34); The Science and Technology Association of Shaanxi (2021PSLK31); The Scientific and Technological Achievements Transformation Project from the Science and Technology Commission of Shaanxi (2018HJCG‐08); the Epidemiological Investigation of Spinal Tuberculosis in Shaanxi Province(20YXYJ0011(3)).
